# *Lactifluusbicapillus* (Russulales, Russulaceae), a new species from the Guineo-Congolian rainforest

**DOI:** 10.3897/mycokeys.45.29964

**Published:** 2019-01-28

**Authors:** Eske De Crop, Jonas Lescroart, André-Ledoux jouonkou, Ruben De Lange, Kobeke Van de Putte, Annemieke Verbeken

**Affiliations:** 1 Research Group Mycology, Department of Biology, Ghent University, Ghent, Belgium Ghent University Ghent Belgium; 2 Department of Biological Sciences, Faculty of Sciences, University of Bamenda, Cameroon University of Bamenda Bamenda Cameroon

**Keywords:** Ectomycorrhizal fungi, *Gilbertiodendron, Lactarius*, phylogeny, taxonomy, tropical Africa, *
Uapaca
*

## Abstract

The milkcap genus *Lactifluus* is one of the most common ectomycorrhizal genera within Central African rainforests. During a field trip to the Dja Biosphere Reserve in Cameroon, a new *Lactifluus* species was found. Molecular and morphological analyses indicate that the species belongs to LactifluussectionXerampelini and we formally describe it here as *Lactifluusbicapillus***sp. nov.**

## Introduction

Rainforests occur in Central Africa and form the main vegetation type in the Guineo-Congolian region (White 1983). Large parts of southern Cameroon and northern Gabon are covered by rainforest, characterised by high humidity, closed canopies, and competition for light in the understory. Common tree species within these rainforests, such as the Dja Biosphere Reserve, include ectomycorrhizal (ECM) species from the Phyllanthaceae (e.g. *Uapaca* spp. Baill.) and the Fabaceae (i.e. *Gilbertiodendrondewevrei* (De Wild.) J.Léonard) (Sonké and Couvreur 2014). *Uapaca* species mainly occur mixed with other tree species, whereas *G.dewevrei* forms more or less monodominant stands, mixed with an occasional *Uapaca* species. These trees are typical hosts for ECM fungi and Russulaceae have been repeatedly recorded as associated with these trees (Verbeken and Walleyn 2010; De Crop et al. 2016; Delgat et al. 2017; T.W. Henkel pers. comm.).

Within Central African rainforests, the ECMRussulaceae genera *Russula* Pers. and *Lactifluus* (Pers.) Roussel are abundant (Douanla-Meli and Langer 2009; Verbeken and Buyck 2002; Verbeken et al. 2008; Verbeken and Walleyn 2010). The milkcap genus *Lactifluus* is mainly distributed in the tropics (De Crop et al. 2017). It is a species-rich genus with about 160 species distributed worldwide, of which the majority is found in tropical Asia (Le et al. 2007b; Stubbe et al. 2010; Van de Putte et al. 2010), tropical Africa (Van de Putte et al. 2009; Verbeken and Walleyn 2010; De Crop et al. 2012, 2016; Maba et al. 2014, 2015a, b; Delgat et al. 2017; De Lange et al. 2018) and the Neotropics (Henkel et al. 2000; Miller et al. 2002; Smith et al. 2011; Sá et al. 2013; Sá and Wartchow 2013). The genus is relatively understudied and many species remain undescribed due to this mainly tropical distribution. Furthermore, the genus is known for its many species complexes with morphologically cryptic species (Stubbe et al. 2010; Van de Putte et al. 2010, 2012; De Crop et al. 2014; De Crop et al. 2017).

About 20 *Lactifluus* species are known from the rainforests of Central Africa (Verbeken and Walleyn 2010). The actual diversity is expected to be higher for several reasons: (i) the ECM flora is present in most parts of the tropical African rainforest, (ii) most countries in the region are understudied due to difficult political situations or challenging sampling conditions, (iii) seasonality in the rainforest is less pronounced, which makes it difficult to assess the exact fruiting period of these fungi and the fruiting of fungi can be missed during short sampling periods, and (iv) *Lactifluus* is known for its morphologically cryptic diversity with several species complexes occurring. Traditional species descriptions were often based on morphology and this morphologically cryptic diversity makes it difficult to correctly assess the number of species based on morphology alone.

During fieldwork in Cameroon in 2012 and 2014, several *Lactifluus* specimens were found morphologically resembling yet different from the described species within L.subg.Pseudogymnocarpi (Pacioni & Lalli) De Crop. The phylogenetic results of De Crop et al. (2017), based on four nuclear genes, revealed that this species is new to science. A preliminary microscopic study confirmed the deviating morphology of the Cameroonian collections and a more detailed study of all available material was initiated. In this study, molecular and morphological examinations were performed, the collections were compared with closely related species, and a new species, *Lactifluusbicapillus*, was described based on these results.

## Methods

### Sampling

Sampling expeditions in Cameroon were carried out in May 2012 and May 2014, in the Guineo-Congolian rainforest of the Dja Biosphere Reserve (East Region of Cameroon), mainly in the vicinity of Somalomo and Lomié. During each expedition, four collections were made of an unknown and putative new milkcap species with characteristics of L.subg.Pseudogymnocarpi. The collections were found in either monodominant stands of *Gilbertiodendrondewevrei*, or mixed stands with *Uapacaguineensis* Müll. Arg., *U.acuminata* (Hutch.) Pax & K. Hoffm., and *U.paludosa* Aubrév. & Leandri as the main ECM hosts. Specimens were dried using a field drier and candles. The studied collections were deposited in the fungal herbarium of Ghent University (**GENT**).

### Morphology

Macroscopic features were all based on fresh material described in the field. Colour codes refer to Kornerup and Wanscher (1978). Microscopic features were studied from dried material. Morphological terminology followed Verbeken and Walleyn (2010). Elements of the pileipellis and hymenium were mounted in Congo Red in L4. Sections of the pileipellis and stipitipellis were first mounted in 10% KOH to enhance cell expansion and then mounted in Congo Red dissolved in water. Basidium length excludes sterigmata length. Measurements are given as MIN–MAX, except for basidiospores. Basidiospores were measured in side view in Melzer’s reagent, excluding the ornamentation, and measurements are given as described in Nuytinck and Verbeken (2005): (MIN) [Ava −2 × SDa] – *Ava* – *Avb* – [Avb + 2 × SDb] (MAX), in which Ava/b = lowest/highest mean value for the measured collections, SDa/b = standard deviation of the lowest/highest mean value. MIN/MAX = lowest/highest value measured and only given when they exceed [Ava −2 × SDa] or [Avb + 2 × SDb] respectively. Q stands for 'quotient length/width' and is given as MINQ – *Qa* – *Qb* – MAXQ, in which Qa/b = lowest/highest mean quotient for the measured specimens, MIN/MAXQ = minimum/maximum value over the quotients of all available measured basidiospores. Line drawings were made with the aid of a drawing tube at the original magnifications: 6000 × for basidiospores (Zeiss axioscop 2 microscope), 1000 × for individual elements and sections (Olympus CX31 microscope).

### Phylogenetic analysis

DNA was extracted using the CTAB extraction protocol described in Nuytinck and Verbeken (2003). Protocols for PCR amplification follow Le et al. (2007a). Two nuclear markers that were previously shown to be informative within this subgenus (De Crop et al. 2017) were used: (1) the internal transcribed spacer region of ribosomal DNA (ITS), comprising the ITS1 and ITS2 spacer regions and the ribosomal gene 5.8S, using primers ITS-1F and ITS4 (Gardes and Bruns 1993; White et al. 1990) and (2) a part of the ribosomal large subunit 28S region (LSU), using primers LR0R and LR5 (Moncalvo et al. 2000).

PCR products were sequenced using an automated ABI 3730 XL capillary sequencer (Life Technology) at Macrogen. Forward and reverse reads were assembled into contigs and edited where needed with the SEQUENCHER v. 5.0 software (Gene Codes Corporation, Ann Arbor, MI, USA).

A dataset was constructed, containing sequences of these recent collections, together with sequences of L.subg.Pseudogymnocarpi extracted from the dataset of De Crop et al. (2017). Furthermore, sequences were compared to sequences in the Unite database using Blastn (Abarenkov et al. 2010). One environmental sequence was found within the same Species Hypothesis and was added to the dataset. The outgroup consisted of four species of L.subg.Lactifluus (Table 1).

**Table 1.** Specimens and GenBank accession numbers of DNA sequences used in the molecular analyses. The arrangement of the subgenera and sections in the table follows their position in the concatenated phylogeny of the genus *Lactifluus* (Fig. 1).

Species	Voucher collection (herbarium)	Country	ITS accession no.	LSU accession no.
**Genus *Lactifluus***				
** Lactifluus subg. Pseudogymnocarpi **				
** Lactifluus sect. Pseudogymnocarpi **				
L. cf. longisporus	AV 11-025 (GENT)	Tanzania	KR364054	KR364181
L. cf. pseudogymnocarpus	AV 05-085 (GENT)	Malawi	KR364012	KR364139
L. cf. pumilus	EDC 12-066 (GENT)	Cameroon	KR364067	KR364196
* L. gymnocarpoides *	JD 885 (BR)	Congo	KR364074	KR364203
	AV 05-184 (GENT)	Malawi	KR364024	KR364151
* L. hygrophoroides *	AV 05-251 (GENT)	North America	HQ318285	HQ318208
* L. longisporus *	AV 94-557 (Isotype, GENT)	Burundi	KR364118	KR364244
* L. luteopus *	AV 94-463 (Isotype, GENT)	Burundi	KR364119	None
* L. medusae *	EDC 12-152 (GENT)	Cameroon	KR364069	KR364198
* L. pseudoluteopus *	FH 12-026 (GENT)	Thailand	KR364084	KR364214
* L. rugatus *	EP 1212/7 (LGAM-AUA)	Greece	KR364104	KR364235
* L. sudanicus *	AV 11-174 (Isotype, GENT)	Togo	HG426469	KR364186
** Lactifluus sect. Xerampelini **				
*L.bicapillus* sp. nov.	EDC 12-176 (GENT)	Cameroon	KR364070	KR364199
	EDC 12-174 (GENT)	Cameroon	MH549201	MH549201
	EDC 14-245 (GENT)	Cameroon	MH549204	MH549204
	EDC 12-169 (GENT)	Cameroon	MH549200	MH549200
	EDC 14-249 (Holotype, GENT)	Cameroon	MH549203	MH549203
	EDC 14-284 (GENT)	Cameroon	KX499395	None
	EDC 14-238 (GENT)	Cameroon	MH549202	MH549202
	EDC 12-071 (GENT)	Cameroon	KX499396	KX622762
	L6470/Gab40 (env. seq.)	Gabon	FR731875	None
L. cf. pseudovolemus	ADK 2927 (GENT)	Benin	KR364113	KR364243
* L. goossensiae *	AB 320 (GENT)	Guinea	KR364132	KR364252
* L. kivuensis *	JR Z 310 (Holotype, GENT)	Congo	KR364027	KR364154
* L. rubiginosus *	JD 959 (BR)	Congo	KR364081	KR364210
	BB 3466 (Holotype, BR)	Zambia	KR364014	KR364250
* L. persicinus *	EDC 12-001 (Holotype, GENT)	Cameroon	KR364061	KR364190
* L. xerampelinus *	TS 1116 (Isotype, GENT)	Tanzania	KR364039	KR364166
**Clade 8**				
*L.* sp.	JN 2011-012 (GENT)	Vietnam	KR364045	KR364171
	TENN 065929 (TENN)	North America	KR364102	KR364233
* L. armeniacus *	EDC 14-501 (Isotype, GENT)	Thailand	KR364127	None
* L. volemoides *	TS 0705 (Holotype, H)	Tanzania	KR364038	KR364165
** Lactifluus sect. Aurantiifolii **				
* L. aurantiifolius *	AV 94-063 (Isotype, GENT)	Burundi	KR364017	KR364144
** Lactifluus sect. Rubroviolascentini **				
L. aff. rubroviolascens	EDC 12-051 (GENT)	Cameroon	KR364066	KR364195
* L. carmineus *	AV 99-099 (Holotype, GENT)	Zimbabwe	KR364131	KR364251
* L. denigricans *	EDC 11-218 (GENT)	Tanzania	KR364051	KR364178
* L. kigomaensis *	AV 11-006 (Holotype, GENT)	Tanzania	KR364052	KR364179
* L. subkigomaensis *	EDC 11-159 (GENT)	Tanzania	KR364050	KR364177
** Lactifluus sect. Polysphaerophori **				
* L. pegleri *	PAM/Mart 12-091 (LIP)	Martinique	KP691416	KP691425
*L.* sp.	RC/Guy 09-036 (LIP)	French Guiana	KJ786645	KJ786550
	MR/Guy 13-145	French Guiana	KJ786691	KJ786595
	MCA 3937 (GENT)	Guyana	KR364109	KR364240
* L. veraecrucis *	M 8025 (Holotype, ENCB)	Mexico	KR364112	KR364241
** Lactifluus subg. Lactifluus **				
** Lactifluus sect. Lactifluus **				
*L.corrugis* s.l.	AV 05-392 (GENT)	North America	JQ753822	KR364143
* L. versiformis *	AV-KD-KVP 09-045 (Holotype, GENT)	India	JN388967	JN389031
* L. vitellinus *	KVP 08-024 (GENT)	Thailand	HQ318236	HQ318144
* L. volemus *	KVP 11-002 (GENT)	Belgium	JQ753948	KR364175

Sequences were aligned using the online version of the multiple sequence alignment program MAFFT v. 7 (Katoh and Standley 2013), using the E-INS-I strategy. Trailing ends of the alignment were trimmed and sequences were manually edited when necessary in MEGA 6 (Tamura et al. 2013). The alignment can be acquired from the first author and TreeBASE (S22916, http://purl.org/phylo/treebase/phylows/study/TB2:S22916).

Sequence data were divided into the following partitions: partial 18S, ITS1, 5.8S, ITS2 and partial 28S. Maximum likelihood (ML) analyses were conducted with RAxML v. 8.0.24 (Stamatakis 2014), where a ML analysis was combined with the Rapid Bootstrapping algorithm with 1000 replicates under the GTRCAT option (Stamatakis et al. 2008). All analyses were performed on the CIPRES Science Gateway (Miller et al. 2010).

## Results

**Figure 1.**
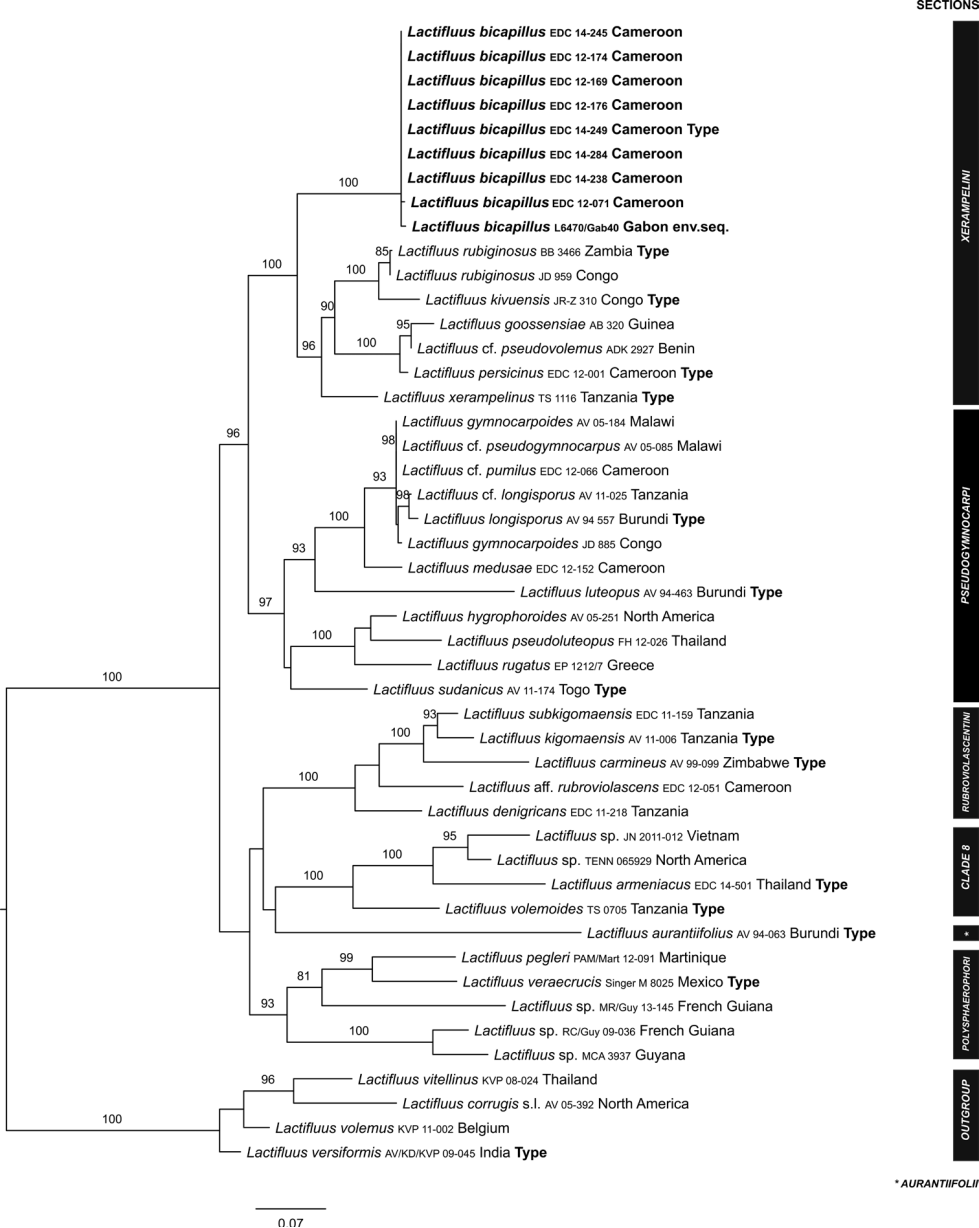
Overview Maximum Likelihood tree of Lactifluussubg.Pseudogymnocarpi, based on concatenated ITS and LSU sequence data. Sequences of the here described species *Lactifluusbicapillus* are written in bold. Maximum Likelihood bootstrap values > 70 are shown. Numbers of undescribed sections refer to De Crop et al. (2017).

Our molecular results show that the recently collected specimens form a well-supported monophyletic clade within Lactifluussubg.Pseudogymnocarpi, L.sect.Xerampelini (Fig. 1). The species is sister to a well-supported clade of all other species within this section, with *L.xerampelinus* (Karhula & Verbeken) Verbeken being its closest relative. Morphological and ecological data confirm that these collections are different from all other species in L.sect.Xerampelini, therefore the new species is described here as *Lactifluusbicapillus* sp. nov.

### Taxonomy

#### Lactifluus bicapillus

Taxon classificationFungiRussulalesRussulaceae

Lescroart & De Crop

827400

Figs 2 , 3 , 4

##### Diagnosis.

*Lactifluusbicapillus* differs from *L.xerampelinus* by its yellowish-orange to dark red cap, fertile lamella edge, a lampropalisade with two types of terminal elements as pileipellis type, and a distribution in the Guineo-Congolian rainforest.

##### Holotype.

CAMEROON. East Region, Haut-Nyong division, Somalomo subdivision, Dja Biosphere Reserve, alt. ca 640 m, 3°21.83'N, 12°44.18'E, rainforest with *Uapacapaludosa* and *U.guineensis*, 14 May 2014, leg.: De Crop & Verbeken, EDC 14-249 (GENT!).

**Basidiocarps** medium-sized. **Pileus** 34–79 mm in diameter, firm, infundibuliform to deeply infundibuliform, planoconvex with central depression when younger; margin involute when juvenile, becoming inflexed up to reflexed when older; edge entire, sometimes eroded when older; surface felty to chamois leather-like, often slightly pruinose in the centre, often grooved, concentrically wrinkled, in young specimens completely velutinous and somewhat translucent; rubiginous (7D6–7) in centre, becoming paler and more orange towards the margin (6C5–6 to 5A5–6); young specimens dark reddish or burgundy in centre, to bright orange or yellow at the margin (8F6 to 7B6, to 6A5, 4AG); secondary velum absent. **Stipe** 16–39 × 6–12 mm, cylindrical to slightly tapering downwards, often laterally curved near the base, central to eccentric insertion to pileus, entire or bruised appearance, sometimes with white flocks near the base; surface smooth and felty, sometimes pruinose, yellowish orange (5AB5–6), becoming slightly paler and more yellow near the base and/or lamellae (5A4–5). **Lamellae** intervenose, transvenose, sparingly bifurcating; attachment adnate to decurrent with some lamellae forming a small tooth; juveniles not brittle, rather thin, older specimens brittle to very brittle, thick to very broad; edge entire and concolourous; distant, 3–5 + 6–9 L+l/cm, between 2 lamellae often 3 lamellulae, with regular short long-short pattern; creamy yellow (3A2) to yellowish orange (4A4). **Context** white, with a faint yellow tinge, colour not changing when cut, but in 1 collection (EDC 14-238) becoming brown when damaged, rather solid and full, smell sweet or not distinct, taste mild. **Latex** white, somewhat astringent, rather abundant, becomes less abundant and more watery with age, mild, colour rarely changing brownish when isolated. **Chemical reactions** unchanging with Fe_2_SO_4_; context faint blue after 5 sec. with guaiac.

**Basidiospores** [6.2]–7.3–7.9–[9.6](10.3) × [4.6]–5.5–5.9–[6.8] μm; ellipsoid, with Q = (1.22)1.31–1.39(1.51); ornamentation amyloid, composed of low ridges and warts, up to 0.2 μm high, forming an incomplete to complete reticulum; plage inamyloid or centrally amyloid. **Basidia** 43–62 × 8–12 μm, rather long, narrowly subclavate, 1-, 2- or 4-spored; content oleiferic. **Sterile elements** abundant, 19.5–40 × 3.5–5.5 μm, not emergent, cylindrical, septate with clamp-like bulges under the septum, with rounded apex. **Pleurocystidia** absent. **Pleuropseudocystidia** very scarce in mature specimens, abundant in young specimens, narrowly and irregular cylindrical to flexuose, 3.3–4.6 μm diam., not emerging, apex obtuse, oleiferic content. **Lamellae-edge** fertile, consisting of basidioles with some basidia. Marginal cells absent. **Hymenophoral trama** cellular, with sphaerocytes and abundant lactifers. **Pileipellis** a lampropalisade, up to 275 μm thick; terminal elements of two types, without transitional forms: the first type long and slender, thick-walled and often septate, with a wide base, up to 7 μm, and growing thinner towards the apex, down to 1–2 μm, length 52–92 μm, often narrowing rather abruptly, and twisted; the second type short and broad, also thick-walled and often septate, not specifically narrower towards the apex, often twisted, 20–44 × 5–7 μm; subpellis composed of mostly rounded cells. **Stipitipellis** similar to pileipellis but not as thick; terminal elements of the long type 52–75 × 5–7 μm; terminal elements of the short type 22–29 × 5–7 μm. **Clamp-connections** absent.

**Figure 2.**
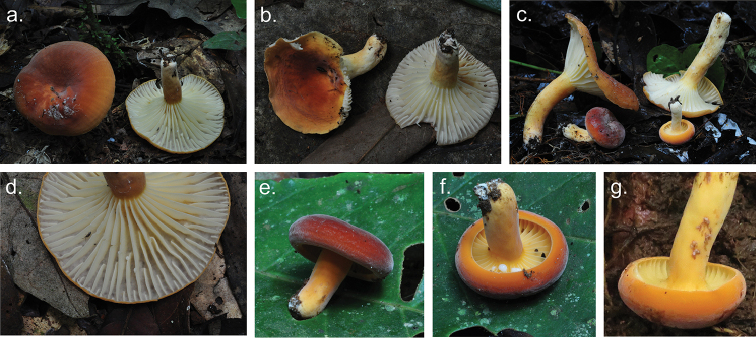
Basidiomata of *Lactifluusbicapillus*. **a–c** Basidiomata of *Lactifluusbicapillus* (EDC 12-176, EDC 12-174, holotype EDC 14-249 resp.) **d** Detail of lamellae (EDC 14-176), e) young specimen (EDC 12-169) **f** Detail of latex (EDC 12-169) **g** Detail of brown colour change of the latex (EDC 14-238) (photographs **a–f** by E. De Crop, **g** by A. Verbeken).

**Figure 3.**
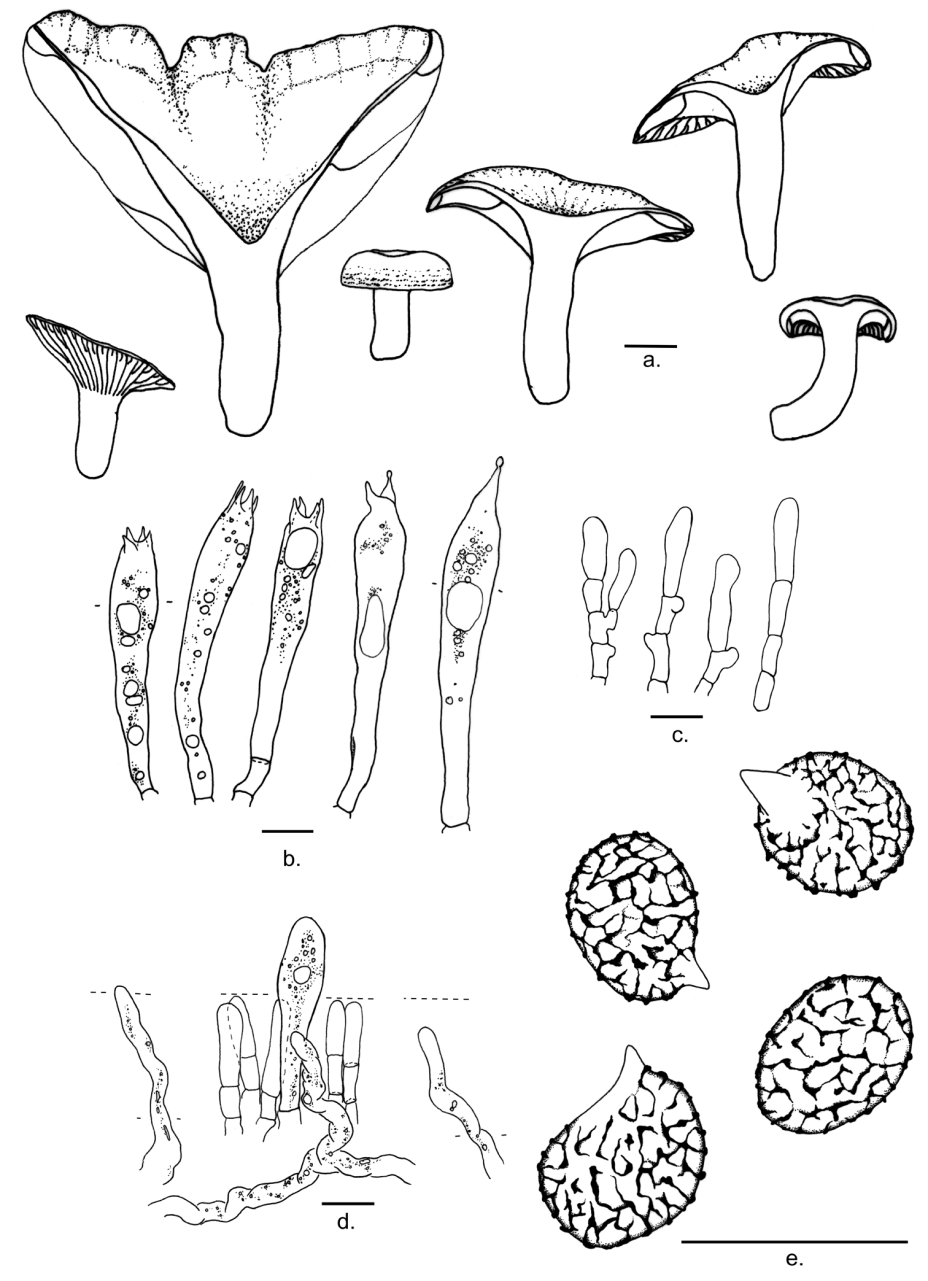
Microscopic features of *Lactifluusbicapillus***a** Basidiocarps (from EDC 12-071, EDC 12-169, EDC 12-174, EDC 12-176, and EDC 14-249) **b** Basidia (from EDC 12-071, and EDC 14-249) **c** Sterile elements from the hymenium (from EDC 12-169) **d** Pleuropseudocystidia (from EDC 12-169) **e** Basidiospores (from EDC 14-249). Illustrations by E. De Crop, J. Lescroart and A. Verbeken. Scale bar: 10 μm.

**Figure 4.**
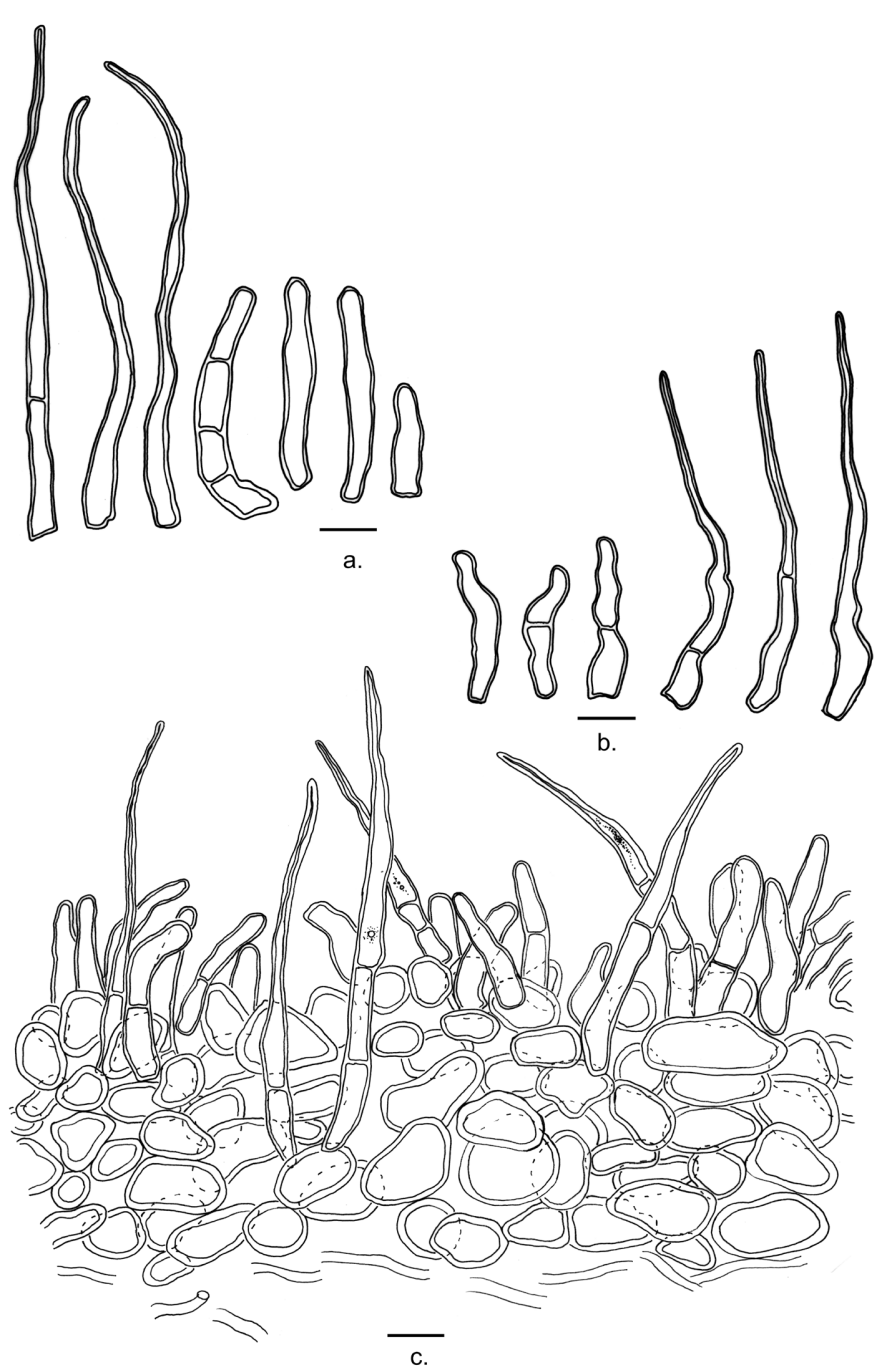
Microscopic features of *Lactifluusbicapillus* (continued) **a** Terminal elements of the pileipellis (from EDC 12-071) **b** Terminal elements of the stipitipellis (from EDC 12-176) **c** Section through the pileipellis (from holotype EDC 14-249). Illustrations by E. De Crop and J. Lescroart. Scale bar: 10 μm.

##### Distribution.

Known from Cameroon and Gabon.

##### Ecology.

Guineo-Congolian rainforest, scattered on forest floor under *Gilbertiodendrondewevrei*, *Uapacaguineensis*, *U.acuminata*, and *U.paludosa*.

##### Etymology.

A combination of ‘bi’ and ‘capillus’, referring to the two types of terminal elements in the pileipellis and stipitipellis.

##### Conservation status.

Unknown.

##### Specimens examined.

Cameroon. East Region, Haut-Nyong division, Somalomo subdivision, Koulou village, alt. ca 650 m, 3°23.61'N, 12°44.50'E, rainforest, *Gilbertiodendrondewevrei*, *Uapacaguineensis*, *U.acuminata*, 15 May 2012, De Crop, EDC 12-071 (GENT); East Region, Haut-Nyong division, Lomié subdivision, Bosquet village, alt. ca 610 m, 3°07.82'N, 13°53.36'E, rainforest with many *Uapaca* trees, on a riverbank, *Uapacaguineensis*, 24 May 2012, De Crop, EDC 12-169 (GENT); Ibidem, *Gilbertiodendrondewevrei*, De Crop, EDC 12-174 (GENT); Ibidem, *Uapacaguineensis*, EDC 12-176 (GENT); East Region, Haut-Nyong division, Somalomo subdivision, Dja Biosphere Reserve, alt. ca 650 m, 3°21.90'N, 12°44.15'E, rainforest, *Uapacapaludosa, U.guineensis*, 14 May 2014, De Crop & Verbeken, EDC 14-238 (GENT); Ibidem, alt. ca 640 m, 3°21.83'N, 12°44.18'E, De Crop & Verbeken, EDC 14-249 (GENT); Ibidem, alt. ca 650 m, 3°19.87'N, 12°45.42'E, rainforest, near the river, *Uapaca* sp., 17/05/2014, De Crop & Verbeken, EDC 14-284 (GENT).

## Discussion

*Lactifluusbicapillus* is recognized in the field by its yellowish-orange to dark-red cap, a concolourous or somewhat paler stipe, yellow lamellae, and unchanging white latex. *L.bicapillus* is placed in L.subg.Pseudogymnocarpi, L.sect.Xerampelini. Species in this section are characterized by yellowish-orange to reddish-brown caps, a palisade-like structure as pileipellis, the absence of true pleurocystidia, and generally low ornamented basidiospores (not higher than 0.2 μm) ranging from verrucose to almost completely reticulate (De Crop et al. 2017). *Lactifluusbicapillus* perfectly concurs with these morphological characteristics, providing additional support for its placement in L.sect.Xerampelini.

**Figure 5.**
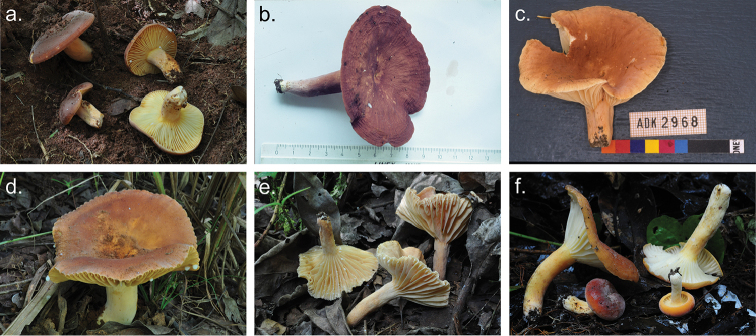
Basidiomata of described species of Lactifluussect.Xerampelini**a***L.xerampelinus* (EDC 11-113) **b**L.*kivuensis* (JR Z 233) **c**L.cf.pseudovolemus (ADK 2968) **d***L.rubiginosus* (EDC 11-120) **e***L.persicinus* (EDC 12-001, *holotypus*) **f***L.bicapillus* (EDC 14-249, holotypus) (photographs a, **d–f** by E. De Crop, **b** by J. Rammeloo and **c** by A. De Kesel).

Lactifluussect.Xerampelini is exclusively known from Africa and contains six described species (Fig. 5): *L.goossensiae* (Beeli) Verbeken, *L.kivuensis* (Verbeken) Verbeken, *L.persicinus* Delgat & De Crop, *L.pseudovolemus* (R. Heim) Verbeken, *L.rubiginosus* (Verbeken) Verbeken, and *L.xerampelinus* (Verbeken and Walleyn 2010; Delgat et al. 2017).

*Lactifluusbicapillus* differs in ecology from all but one species of L.sect.Xerampelini. Species from this section occur in woodlands, gallery forests and rainforests (Verbeken and Walleyn 2010). *Lactifluusxerampelinus* and *L.rubiginosus* are found in miombo woodland in East Africa, while *L.goossensiae* is known from both Sudanian woodland and Central African gallery forests. *Lactifluuspersicinus* and *L.pseudovolemus* occur in West African gallery forests. Both *L.kivuensis* and *L.bicapillus* are found in the Guineo-Congolian rainforest, associated with *Gilbertiodendrondewevrei* and *Uapaca* species.

Macroscopically, *L.bicapillus* differs from the other species of this section by a combination of bright cap colours, which vary from dark red to bright orange near the edge, cream white lamellae and pale yellow-orange stipe colours in adult basidiocarps (Fig. 5).

All species from L.sect.Xerampelini have ellipsoid to elongate basidiospores, with amyloid ornamentation composed of very low warts and ridges (up to 0.2 µm high) that are isolated, aligned or forming an incomplete reticulum. All seven species have long and slender basidia, mostly cylindrical and 4-spored. However, 1- and 2-spored basidia are present in *L.bicapillus*, *L.persicinus*, and *L.pseudovolemus*. True cystidia are absent in all species. Pleuropseudocystidia are scarce in *L.bicapillus*, *L.persicinus*, and *L.kivuensis*, abundant in the other species. These pleuropseudocystidia are occasionally emergent in all species; however, emergent pleuropseudocystidia were not observed in *L.bicapillus*. *Lactifluuspersicinus* and *L.bicapillus* have a fertile lamellar edge, whilst the others have a sterile lamellar edge (or unknown in *L.pseudovolemus* and *L.goossensiae*).

All species of this section have palisade-like structures as pileipellis. *Lactifluusbicapillus*, *L.persicinus*, and *L.goossensiae* have a lampropalisade with thick-walled terminal elements. *Lactifluuspseudovolemus* has a palisade in which the elements of the pileipellis are slightly thickened. *Lactifluuskivuensis*, *L.xerampelinus*, and *L.rubiginosus* have a palisade to trichopalisade, with only thin-walled elements of the pileipellis. Only *Lactifluusbicapillus*, *L.persicinus*, and *L.goossensiae* have terminal elements that are narrow near the apex. Furthermore, *L.bicapillus* is the only species within this section with two types of terminal elements in the pilei- and stipitipellis.

With the finding of *Lactifluusbicapillus*, L.sect.Xerampelini now contains seven described species, all from sub-Saharan Africa. Together with the recently described *L.persicinus* (Delgat et al. 2017), *L.bicapillus* was found during two sampling expeditions in Cameroon. Even though those expeditions only covered a small area of the Guineo-Congolian rainforest and gallery forests, we collected at least five species new to science (De Crop et al. 2017). This highlights the large *Lactifluus* diversity in Africa, with many areas still unexplored and probably many new species still to be found.

## Supplementary Material

XML Treatment for Lactifluus bicapillus
